# Mature Tertiary Lymphoid Structures Indicate Good Chemotherapy Response and Prognosis in Advanced Colorectal Cancer

**DOI:** 10.1002/ags3.70142

**Published:** 2025-12-02

**Authors:** Nobuhiro Hosoi, Chika Komine, Gendensuren Dorjkhorloo, Takuya Shiraishi, Takuhisa Okada, Akihiko Sano, Makoto Sakai, Takehiko Yokobori, Ken Shirabe, Hiroshi Saeki

**Affiliations:** ^1^ Department of General Surgical Science Gunma University Graduate School of Medicine Maebashi Japan; ^2^ Division of Integrated Oncology Research Gunma University, Initiative for Advanced Research (GIAR) Maebashi Japan

**Keywords:** advanced colorectal cancer, immune cell infiltration, immune status, systemic chemotherapy, tertiary lymphoid structures

## Abstract

**Background:**

Tertiary lymphoid structures (TLS), clusters of immune cells including B and T cells, are involved in anti‐tumor immunity and correlate with a favorable prognosis in several malignancies, but their association with systemic chemotherapy is unknown. We investigated the role of TLS as a biomarker for predicting chemotherapy efficacy in advanced colorectal cancer (CRC).

**Methods:**

We analyzed 78 CRC cases with metastatic or recurrent lesions, in which untreated resected primary tumors were available before chemotherapy. TLS maturity, defined by the presence of CD23^+^ B cells in germinal centers, was assessed by immunohistochemistry and evaluated in relation to clinicopathological features, treatment response, tumor‐infiltrating lymphocytes, and prognosis. In 11 cases with resected metastatic lesions, tumor‐infiltrating lymphocytes were analyzed to explore the relationship between TLS maturity in primary tumors and immune status at distant sites.

**Results:**

Mature TLS‐negative groups had poorer chemotherapy sensitivity, progression‐free survival, and overall survival. Multivariate analysis confirmed TLS maturity as an independent predictor of both therapeutic response and survival outcomes in this cohort. Mature TLS was associated with higher CD3^+^ and CD8^+^ T‐cell infiltration in both primary and metastatic tumors.

**Conclusion:**

Mature TLS may contribute to favorable chemotherapeutic efficacy through immune activity. TLS maturity in primary tumors may represent a novel biomarker for predicting systemic chemotherapy response and immune status across primary and metastatic sites in advanced CRC.

## Graphical abstract

In advanced colorectal cancer, tumors with mature tertiary lymphoid structures (TLS) exhibited abundant infiltration of CD3‐ and CD8‐positive lymphocytes in both primary and metastatic sites, indicating an activated immune response. The presence of mature TLS was also associated with favorable chemotherapy sensitivity and improved prognosis.

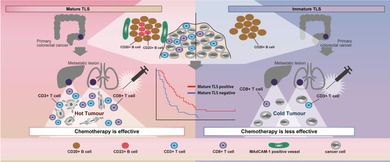

**Abbreviations**
CR: complete response
CRC: colorectal cancer
DAB: 3,3′‐diaminobenzidine tetrahydrochloride
HEVs: high endothelial venules
IARC: International Agency for Research on Cancer
OS: overall survival
PD: progressive disease
PFS: progression‐free survival
PR: partial response
SD: stable disease
TLS: tertiary lymphoid structure

## 1 Introduction

Colorectal cancer (CRC) is the second leading cause of cancer‐related death and the third most commonly diagnosed cancer worldwide [1]. Surgical resection remains the primary treatment option for resectable lesions, including distant metastases, and the efficacy of neoadjuvant chemotherapy for metastatic lesions has been actively investigated [2, 3]. In contrast, systemic chemotherapy is the mainstay of treatment for unresectable CRC and has undergone remarkable progress in recent years [4]. Recently, the development of immune checkpoint inhibitors has broadened the therapeutic landscape across various cancer types, including CRC, contributing to improved clinical outcomes [5, 6]. Nevertheless, despite these therapeutic advances, the prognosis of treatment‐resistant CRC remains poor. Therefore, developing reliable biomarkers to predict chemosensitivity and patient prognosis is a critical unmet need in clinical practice.

Tertiary lymphoid structure (TLS) is an ectopic lymphoid aggregate composed of immune cells, such as B and T cells, which forms in the peritumoral or inflammatory microenvironment and exerts immune functions analogous to secondary lymphoid organs, including antibody production, cytokine secretion, and antigen presentation [7]. TLS, characterized by clusters of CD20^+^ B cells surrounded by CD3^+^ T cells and containing CD23^+^ germinal centers, is classified as functionally mature [8, 9]. In the tumor microenvironment, mature TLS contributes to anti‐tumor immunity through its roles in antigen presentation and differentiation into plasma cells that produce tumor‐associated antibodies [10]. Moreover, the presence of TLS has been reported to correlate with a favorable prognosis in several malignancies [7].

We previously reported that mature TLS in the primary tumor of patients with locally advanced CRC who underwent curative resection was an independent prognostic factor for postrecurrence survival [11]. As systemic chemotherapy is typically administered after recurrence, we hypothesized that mature TLS in the primary tumor may relate to chemosensitivity, contributing to prolonged postrecurrence survival. However, to our knowledge, no study to date has investigated whether the maturity of TLS in primary tumors is associated with the therapeutic efficacy of systemic chemotherapy in patients with CRC with targetable metastatic or recurrent lesions. Thus, this hypothesis remains to be validated.

This study aimed to clarify the clinical significance of TLS maturity in primary tumors as a predictive biomarker for the efficacy of systemic chemotherapy in CRC. We analyzed 78 CRC cases with metastatic or recurrent lesions, in which untreated resected primary tumors were available before chemotherapy. TLS maturity, defined by the presence of CD23^+^ B cells in germinal centers, was assessed by immunohistochemistry and evaluated in relation to clinicopathological features, treatment response, and prognosis. Specifically, we investigated the association between mature TLS, the presence of MAdCAM‐1–positive vessels that mediate lymphocyte recruitment, and intratumoral immune cell infiltration, including T cells (CD3, CD8, FOXP3), macrophages (CD86, CD163), and CD20^+^ B cells. Furthermore, in 11 cases with available metastatic lesions, intratumoral CD3^+^ and CD8^+^ T‐cell infiltration in metastatic tumors were analyzed to assess the relationship between TLS maturity in primary tumors and immune cell composition at distant sites targeted by systemic chemotherapy.

## 2 Methods

### 2.1 Patients and Samples

This retrospective study was approved by the ethics committee of the Graduate School of Medicine, Gunma University (protocol number: HS2024‐072). Informed consent was obtained through an opt‐out method on the website. The study included 78 resected primary lesions (*n* = 78) and resected metastatic lesions (*n* = 11) from 78 patients with CRC who initiated their first chemotherapy regimen between January 2012 and December 2019. Of the 78 cases, 43 were noncurative resection cases, and 35 were recurrence cases. Noncurative resection cases were defined as patients who presented synchronous metastases at diagnosis but underwent initial resection of the primary tumor due to complications such as bleeding or obstruction, followed by the introduction of chemotherapy. Recurrence cases were defined as patients who underwent curative resection of the primary tumor but subsequently developed recurrence, for which chemotherapy was administered. Among the 78 patients, 11 developed recurrent diseases after resection of both the primary and metastatic lesions and subsequently received systemic chemotherapy. The 11 metastatic lesions that were resected prior to the initiation of systemic chemotherapy were analyzed as resected metastatic lesions. Cases of chemotherapy dropout within three courses, appendiceal cancer, and anal canal cancer were excluded. Patients who underwent neoadjuvant chemotherapy prior to resection of the primary tumor were also excluded. Accordingly, both the primary and metastatic lesions analyzed in this study had not been modified by chemotherapy, radiotherapy, or other interventions. All clinicopathological data were obtained from clinical and pathological records. The best overall response to first‐line chemotherapy was assessed as complete response (CR), partial response (PR), stable disease (SD), or progressive disease (PD), according to the Response Evaluation Criteria in Solid Tumours version 1.1 [12]. Pathologic stage was classified according to the 8th edition of the TNM classification of the International Union Against Cancer (UICC).

### 2.2 Immunohistochemical Staining

Paraffin‐embedded blocks were cut into 4 μm‐thick sections and mounted on glass slides. Sections were deparaffinized in xylene and dehydrated in alcohol. Endogenous peroxidase activity was inhibited by incubation with 0.3% H₂O₂ in methanol for 30 min at room temperature. After rehydration through a graded series of ethanol treatments, antigen retrieval was performed using Immunosaver (Nisshin EM, Tokyo, Japan) at 98°C–100°C for 45 min. Sections were treated with 0.3% hydrogen peroxide in 100% methanol for 30 min at 20°C–25°C to inhibit endogenous peroxidase activity. Nonspecific binding sites were blocked by incubation with Protein Block Serum‐Free (Dako, Carpinteria, CA, USA) for 30 min. Next, the sections were exposed to primary antibodies in REAL Antibody Diluent (Agilent, Santa Clara, CA, USA) at 4°C for 24 h. These primary antibodies were used for single staining: CD3 (1:1; Ventana, Tucson, AZ, USA; 790–4341), CD8 (1:400; Abcam, Cambridge, UK; ab4055), FOXP3 (1:500; Abcam; ab20034), CD86 (1:400; Cell Signaling Technology, Danvers, MA, USA; CST‐91882S), and CD163 (1:500; Cell Signaling Technology; CST‐93498S). The Histofine Simple Stain MAX‐PO (Multi) Kit (Nichirei, Tokyo, Japan) was used as the secondary antibody at room temperature for 30 min. Chromogen 3,3′‐diaminobenzidine tetrahydrochloride (DAB) was applied as a 0.02% solution containing 0.005% H_2_O_2_ in 50 mM ammonium acetate–citrate acid buffer (pH 6.0).

Nuclear counterstaining was performed using Mayer's hematoxylin solution. Negative control slides were incubated without the primary antibodies, and no detectable staining was observed.

### 2.3 Multi‐Color Immunohistochemistry

After blocking nonspecific binding sites with Protein Block Serum‐Free Reagent (Dako, Carpinteria, CA, USA) for 30 min, the following primary antibodies were applied for multi‐color staining on a single slide: CD20 (1:1; Ventana; 760–2531), CD23 (1:1000; Cell Signaling Technology; CST‐87960S), and MAdCAM‐1 (1:500; Proteintech, Chicago, IL, USA; 66594–1‐Ig).

For DAB detection, the anti‐CD20 antibody was used as the first primary antibody and incubated at 4°C for 24 h. The Histofine Simple Stain MAX‐PO (Multi) Kit (Nichirei) was used as the secondary antibody at room temperature for 30 min. The chromogen DAB (Fujifilm, Tokyo, Japan; 045–22833) was applied as a 0.02% solution containing 0.005% H_2_O_2_ in 50 mM ammonium acetate–citric acid buffer (pH 6.0). After washing with phosphate‐buffered saline (PBS), the slides were incubated in 50 mM citric acid using a microwave for 15 min to inactivate residual antibodies and labeling enzymes. Nonspecific binding sites were then re‐blocked using Protein Block Serum‐Free Reagent (Dako) for 30 min.

For Fast Red detection, the anti‐CD23 antibody was used as the second primary antibody and incubated at 4°C for 24 h. The Histofine Simple Stain AP (R) Kit (Nichirei) was used as the secondary antibody, and Fast Red substrate (Abcam; ab64254) was applied for visualization. After washing with PBS, the same antigen inactivation procedure was performed, followed by an additional blocking step with Protein Block Serum‐Free Reagent (Dako) for 30 min.

For PermaGreen detection, the anti‐MAdCAM‐1 antibody was applied as the third primary antibody and incubated at 4°C for 24 h. The Histofine Simple Stain MAX‐PO (Multi) Kit (Nichirei) was used as the secondary antibody, and PermaGreen substrate (Diagnostic BioSystems; K074) was applied for staining. Nuclear counterstaining was performed with Mayer's hematoxylin solution. Negative control slides were processed in parallel without primary antibodies, and no detectable staining was observed.

### 2.4 Evaluation of TLS and MAdCAM‐1

Sequential sections of surgical specimens were subjected to immunohistochemical staining for CD20, CD23, and MAdCAM‐1. Clusters of CD20^+^ B cells were defined as TLS, and those containing CD23^+^ B cells within a germinal center were classified as mature TLS [8, 9]. Tumor margins were assessed within 1 mm of the invasive front. Cases with at least one mature TLS within this region were categorized as the mature TLS‐positive group [11, 13].

For MAdCAM‐1 evaluation, a 1‐mm tumor margin from the invasive front was assessed, and one TLS was selected per case. If no mature TLS was present, the largest immature TLS was selected. When multiple TLSs were present, mature TLSs were prioritized. If more than one mature TLS met the criteria, the TLS with the largest area was selected. The number of MAdCAM‐1‐positive vessels within and along the periphery of the selected TLS was counted [14].

### 2.5 Image Acquisition and Quantitative Evaluation of Immune Cells

We evaluated tumor‐infiltrating immune cells by immunohistochemical staining for CD3, CD8, FOXP3, CD86, CD163, and CD20. From each tumor section, 36 images covering four representative fields (total area: 9.070624 mm^2^) were captured using a BZ‐X700 microscope (Keyence, Osaka, Japan) [11]. Immune cells were quantified using HALO (Indica Labs, Japan), a semi‐automated image analysis software. Cell density was calculated by dividing the number of positive cells by the analyzed area and expressed as cells per mm^2^.

### 2.6 Statistical Analysis

Statistical analyses were performed using JMP Pro 17 software (SAS Institute Inc., Cary, NC, USA). The chi‐squared test and the Wilcoxon test were used to assess statistical significance for categorical and continuous variables, respectively. Kaplan–Meier curves were constructed to evaluate progression‐free survival (PFS) and overall survival (OS), and differences between groups were assessed using the log‐rank test and the Wilcoxon test. Univariate and multivariate survival analyses for PFS and OS were conducted using Cox proportional hazards models. Univariate and multivariate logistic regression analyses were performed to evaluate the predictive value for therapeutic response (CR and PR). *p* values < 0.05 were considered statistically significant.

## 3 Results

### 3.1 Evaluation of Distribution and Maturity of TLS in Primary Tumors of Metastatic or Recurrent Patients With CRC

**FIGURE 1:**
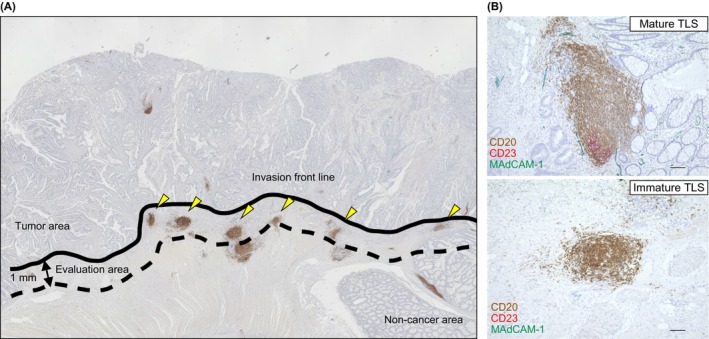
Evaluation of TLS in advanced CRC samples. (A) The evaluation area was defined as 1 mm from the invasion front line. TLS was evaluated within the evaluation area. The yellow arrowhead indicated the evaluated TLS. (B) Representative TLS immunohistochemical staining images are shown. The upper panel shows a representative image of a mature TLS, characterized by a cluster of CD20^+^ B cells with CD23^+^ B cells in the germinal centers. MAdCAM‐1–positive vessels are observed surrounding the TLS. The lower panel shows a representative image of an immature TLS, consisting of a cluster of CD20^+^ B cells without detectable CD23^+^ B cells in the germinal centers. CD20^+^ B cells are stained brown, CD23^+^ B cells are stained red, and MAdCAM‐1–positive vessels are stained green. Images were captured at 100× magnification. Scale bars represent 100 μm.

We defined the peritumoral region as the area extending 1 mm from the invasive front of the tumor tissue to evaluate TLS maturity (Figure 1A). Clusters of CD20^+^ B cells were identified as TLS, and their maturity was determined by the presence of CD23^+^ B cells within germinal centers (Figure 1B). CRC cases with at least one mature TLS in the peritumoral region were classified as mature TLS‐positive. Notably, mature TLS were more frequently surrounded by MAdCAM‐1–positive vessels, which are key mediators of lymphocyte recruitment into tissues, than immature TLS (Figure 1B). Among the 78 CRC cases, 35 (44.9%, 35/78) were classified as mature TLS‐positive and 43 (55.1%, 43/78) as mature TLS‐negative.

### 3.2 Association of Mature TLS With Clinicopathologic Factors and First‐Line Chemotherapeutic Response

Table 1 summarizes the association between mature TLS positivity in primary tumors and various clinicopathologic factors in 78 patients with metastatic or recurrent CRC who received systemic chemotherapy. The mature TLS‐positive group had a significantly higher number of MAdCAM‐1–positive vessels surrounding the TLS (*p* = 0.0002). The mature TLS‐negative group showed a higher proportion of SD + PD, whereas the mature TLS‐positive group exhibited greater sensitivity to first‐line chemotherapy (Table 1). No significant associations were observed between TLS maturity and tumor status, patient characteristics, or molecular profiling status (Table S1).

**TABLE 1:** Patient characteristics.

Characteristics	Mature TLS		*p*
	Negative (*n* = 43)	Positive (*n* = 35)	
Age, years			
65 >	16 (37.2)	16 (45.7)	0.4477
65 ≦	27 (62.8)	19 (54.3)	
Sex (%)			
Female	13 (30.2)	11 (31.4)	0.9094
Male	30 (69.8)	24 (68.6)	
Ethnicity (%)			
Asian	43 (100)	35 (100)	
Tumor location			
Colon	28 (65.1)	21 (60.0)	0.6419
Rectum	15 (34.9)	14 (40.0)	
Disease status			
Noncurative resection cases	25 (58.1)	18 (51.4)	0.5534
Recurrence cases	18 (41.9)	17 (48.6)	
Poorly differentiated component			
Positive	18 (41.9)	20 (57.1)	0.1793
Negative	25 (58.1)	15 (42.9)	
First‐line molecularly targeted drug			
Nothing	11 (25.6)	5 (14.3)	0.2308
VEGF inhibitor	29 (67.4)	24 (68.6)	
EGFR inhibitor	3 (7.0)	6 (17.1)	
First‐line anticancer drug regimen			
CAPOX/SOX/FOLFOX	29 (67.4)	28 (80.0)	0.4421
CAPIRI/IRIS/FOLFIRI	7 (16.3)	3 (8.6)	
TS‐1/Capecitabine	7 (16.3)	4 (11.4)	
Number of MAdCAM‐1 positive vessels (per TLS) (mean (SD))	1.5 (3.1)	4.5 (3.9)	0.0002*
Therapeutic effect			
CR + PR	20 (46.5)	25 (71.4)	0.0267*
PD + SD	23 (53.5)	10 (28.6)	

*Note:* *p < 0.05.

Abbreviations: CAPIRI, capecitabine + irinotecan; CAPOX, capecitabine + oxaliplatin; CR, complete response; EGFR, epidermal growth factor receptor; FOLFIRI, 5‐FU + leucovorin + irinotecan; FOLFOX, 5‐fluorouracil (5‐FU) + leucovorin + oxaliplatin; IRIS, irinotecan + TS‐1; MAdCAM‐1, mucosal vascular addressin cell adhesion molecule 1; *p* value, probability value; PD, progressive disease; PR, partial response; SD, stable disease; SD, standard deviation; SOX, TS‐1 + oxaliplatin; TLS, tertiary lymphoid structures; VEGF, vascular endothelial growth factor.

To evaluate the potential of mature TLS status in primary tumors as a predictive biomarker, we performed univariate and multivariate analyses for favorable therapeutic response, defined as CR or PR, using data from 78 CRC cases. Mature TLS negativity in primary tumors was identified as an independent predictor of poor response to first‐line systemic chemotherapy in patients with metastatic or recurrent CRC (odds ratio = 0.31, 95% confidence interval [CI] = 0.11–0.86, *p* = 0.0242; Table 2).

**TABLE 2:** Univariate and multivariate analyses for predictors of response (CR + PR).

Factors		Univariate		Multivariate	
		Odds ratio (95% CI)	*p*	Odds ratio (95% CI)	*p*
Age, years	65 >	1	0.1019	1	0.1881
	65 ≦	0.45 (0.18–1.17)		0.51 (0.19–1.39)	
Sex	Female	1	0.6746	1	0.7362
	Male	1.23 (0.47–3.24)		1.20 (0.42–3.41)	
Tumor location	Colon	1	0.8984	1	0.6303
	Rectum	1.06 (0.42–2.69)		1.29 (0.45–3.69)	
Disease status	Noncurative resection cases	1	0.3136	1	0.2371
	Recurrence cases	0.63 (0.25–1.55)		0.54 (0.20–1.49)	
Poorly differentiated component	Positive	1	0.6722	1	0.3865
	Negative	1.21 (0.49–2.99)		1.56 (0.57–4.30)	
Mature TLS	Positive	1	0.0288*	1	0.0242*
	Negative	0.35 (0.13–0.90)		0.31 (0.11–0.86)	

*Note:* *p < 0.05.

Abbreviations: Cl, confidence interval; CR, complete response; *p* value, probability value; PR, partial response; TLS, tertiary lymphoid structures.

### 3.3 Prognostic Value of Mature TLS in Primary Tumors of Patients With Advanced CRC Treated With Systemic Chemotherapy

**FIGURE 2:**
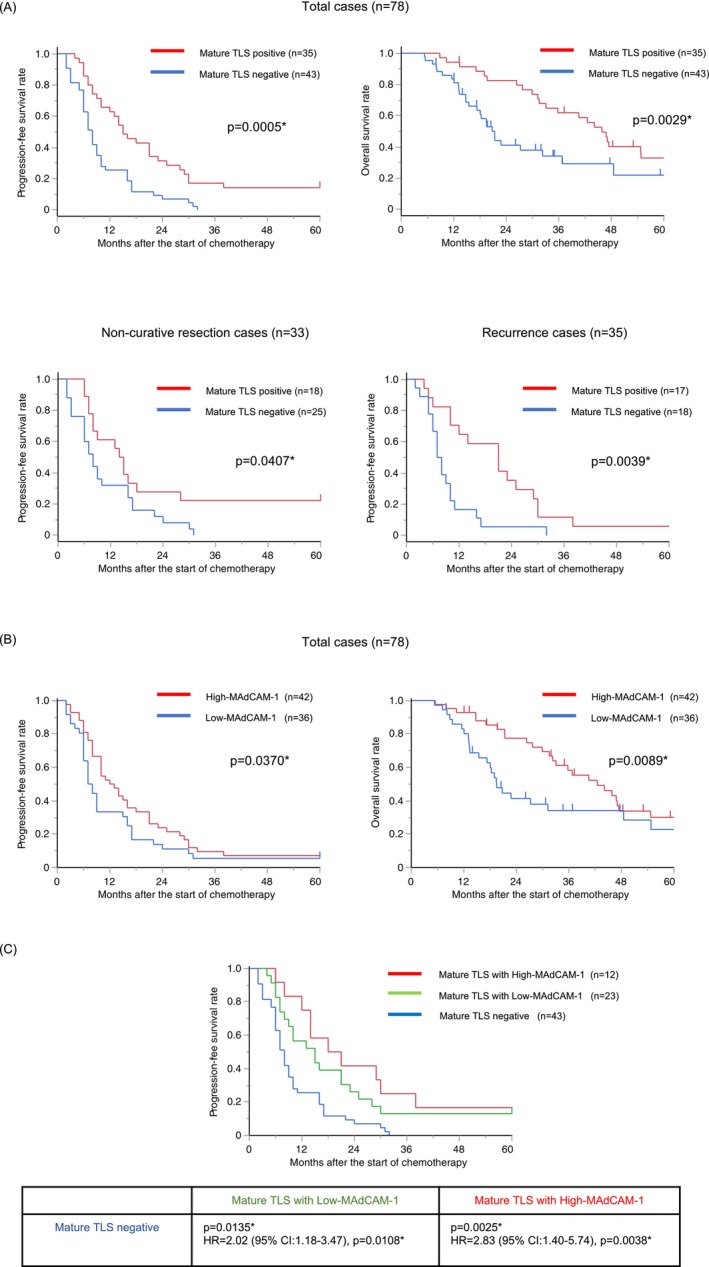
Kaplan–Meier survival curves of patients with advanced CRC stratified by TLS maturity and number of MAdCAM‐1–positive vessels. (A) Kaplan–Meier analyses of PFS in all cases (*p* = 0.0005, upper left panel), OS in all cases (*p* = 0.0029, upper right panel), PFS in noncurative resection cases (*p* = 0.0407, lower left panel), and PFS in recurrence cases (*p* = 0.0039, lower right panel). (B) Kaplan–Meier analyses of PFS in all cases (*p* = 0.0370, left panel), OS in all cases (*p* = 0.0089, right panel). (C) Kaplan–Meier analyses of PFS in the three groups. *p < 0.05; statistical significance was determined using the Wilcoxon test. For the three‐group comparison in (C), Bonferroni correction was applied, and the significance threshold was set at *p* = 0.0167.

The prognostic impact of mature TLS in primary tumors was evaluated using survival analyses of PFS and OS in 78 patients with advanced CRC treated with systemic chemotherapy. The mature TLS‐positive group was significantly associated with longer PFS and longer OS (PFS: *p* = 0.0005, OS: *p* = 0.0029; Figure 2A, top panel). As a subgroup analysis, noncurative resection cases (*n* = 33) and recurrence cases after curative resection (*n* = 35) were analyzed separately. In both subgroups, the mature TLS‐positive group exhibited significantly longer PFS than the mature TLS‐negative group (Figure 2A, bottom panel). Furthermore, a subgroup analysis was conducted based on the site of the target lesion for first‐line chemotherapy. The analysis included patients with liver metastasis (*n* = 52), lung metastasis (*n* = 30), or peritoneal dissemination (*n* = 21; some patients had target lesions in multiple organs). In each target lesion subgroup, the mature TLS‐positive group demonstrated longer PFS than the mature TLS‐negative group (Figure S1).

Table 3 presents the results of univariate and multivariate analyses of PFS and OS using a Cox regression model. The multivariate analysis revealed that mature TLS negativity in primary tumors was an independent predictor of disease progression despite systemic chemotherapy (hazard ratio (HR) = 1.85, 95% CI: 1.09–3.14, *p* = 0.0223) and poor OS (HR = 1.93, 95% CI: 1.29–3.64, *p* = 0.043) in patients with metastatic or recurrent CRC (Table 3).

**TABLE 3:** Univariate and multivariate analyses for progression‐free survival and overall survival.

Factors		Progression‐free survival				Overall survival			
		Univariate		Multivariate		Univariate		Multivariate	
		HR (95% CI)	*p*	HR (95% CI)	*p*	HR (95% CI)	*p*	HR (95% CI)	*p*
Age, years	65 >	1	0.0901	1	0.4375	1	0.5957	1	0.3442
	65 ≦	2.02 (0.89–4.55)		1.21 (0.74–1.98)		0.86 (0.49–41.50)		0.76 (0.42–1.35)	
Sex	Female	1	0.3049	1	0.4462	1	0.4953	1	0.2645
	Male	1.54 (0.67–3.55)		0.82 (0.49–1.37)		1.24 (0.67–2.30)		1.44 (0.76–2.74)	
Tumor location	Colon	1	0.4154	1	0.4159	1	0.1706	1	0.1457
	Rectum	0.82 (0.51–1.32)		0.81 (0.49–1.34)		0.66 (0.36–1.20)		0.63 (0.34–1.17)	
Disease status	Noncurative resection cases	1	0.9637	1	0.8330	1	0.0969	1	0.1220
	Recurrence cases	1.01 (0.64–1.60)		1.05 (0.65–1.70)		0.61 (0.37–1.09)		0.63 (0.35–1.13)	
Poorly differentiated component	Positive	1	0.8313	1	0.5992	1	0.1374	1	0.0513
	Negative	0.95 (0.60–1.51)		1.13 (0.70–1.85)		0.65 (0.60–1.15)		0.57 (0.32–1.00)	
Therapeutic effect	CR + PR + SD	1	< 0.0001*	1	< 0.0001*	1	0.0354*	1	0.2524
	PD	12.06 (5.43–26.77)		9.34 (4.00–21.85)		2.27 (1.06–4.86)		1.65 (0.70–3.86)	
Mature TLS	Positive	1	0.0008*	1	0.0223*	1	0.0195*	1	0.0430*
	Negative	2.28 (1.41–3.70)		1.85 (1.09–3.14)		1.98 (1.12–3.52)		1.93 (1.02–3.64)	

*Note:* *p < 0.05.

Abbreviations: Cl, confidence interval; CR, complete response; *p* value, probability value; PD, progressive disease; PR, partial response; SD, stable disease; TLS, tertiary lymphoid structures.

### 3.4 Relationship Between MAdCAM‐1–Positive Vessels, TLS Maturity, and Prognosis in CRC

The number of MAdCAM‐1–positive vessels was significantly higher in the mature TLS–positive group (*p* = 0.0002; Table 1, Figure 1B).

We then evaluated the prognostic relevance of MAdCAM‐1–positive vessels in 78 patients with advanced CRC who received systemic chemotherapy.

In this cohort, the median number of MAdCAM‐1–positive vessels were two. Accordingly, patients were classified into two groups: High‐MAdCAM‐1 (≥ 2 vessels) and Low‐MAdCAM‐1 (< 2 vessels). Among the 78 cases, 42 (53.8%) were categorized as High‐MAdCAM‐1 and 36 (46.2%) as Low‐MAdCAM‐1.

The High‐MAdCAM‐1 group was significantly associated with prolonged PFS and OS, supporting the prognostic value of MAdCAM‐1–positive vessels (PFS: *p* = 0.0370; OS: *p* = 0.0089; Figure 2B).

Furthermore, we evaluated the prognostic significance of MAdCAM‐1–positive vessels within the Mature TLS–positive group. The third quartile of MAdCAM‐1–positive vessels in this group was six; therefore, patients were classified into three groups: Mature TLS with High‐MAdCAM‐1 (≥ 6 vessels), Mature TLS with Low‐MAdCAM‐1 (< 6 vessels), and Mature TLS negative. In the analysis of PFS, both the Mature TLS with High‐MAdCAM‐1 and the Mature TLS with Low‐MAdCAM‐1 groups showed significantly better outcomes than the Mature TLS negative group. The hazard ratios were as follows: Mature TLS with Low‐MAdCAM‐1 vs. Mature TLS negative (HR = 2.02, 95% CI: 1.18–3.47, *p* = 0.0108), and Mature TLS with High‐MAdCAM‐1 vs. Mature TLS negative (HR = 2.83, 95% CI: 1.40–5.74, *p* = 0.0038). Notably, the HR was higher in comparison between Mature TLS with High‐MAdCAM‐1 and Mature TLS negative (Figure 2C).

### 3.5 Association of TLS Maturity and Intratumoral Immune Cell Infiltration in Primary and Metastatic Tumors of Patients With Metastatic or Recurrent CRC

**FIGURE 3:**
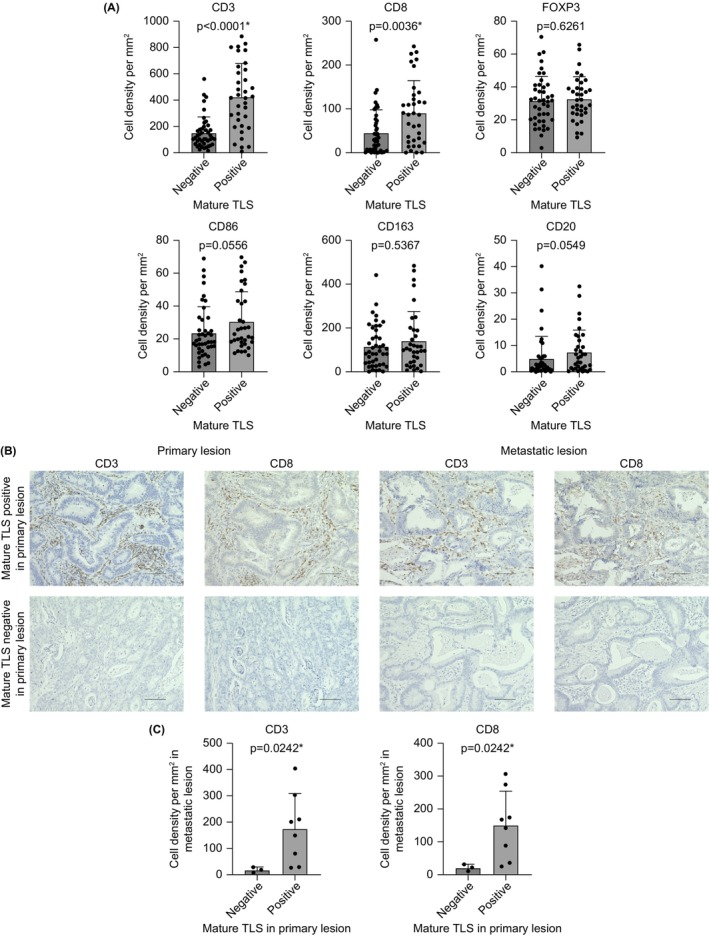
Relationship between mature TLS and intratumoral immune cell infiltration in CRC tissue. (A) Intratumoral infiltration of immune cells (CD3, CD8, FOXP3, CD86, CD163, and CD20) was compared to TLS maturity. (B) Representative immunohistochemical staining images are presented. The upper panel shows CD3 and CD8 staining of mature TLS‐positive primary and metastatic lesions, whereas the lower panel shows CD3 and CD8 staining of mature TLS‐negative primary and metastatic lesions. The metastatic lesions shown are representative images of lung metastases. Images were captured at 200× magnification. Scale bars represent 100 μm. (C) Relationship between TLS maturity in the primary lesion and intratumoral infiltration of CD3^+^ and CD8^+^ cells in metastatic lesions. Among 78 cases in our cohort, metastatic lesions were identified in 11 cases (seven with liver metastasis and four with lung metastasis). Among these 11 cases, eight were mature TLS‐positive in the primary lesion, whereas three were negative for mature TLS. Data are presented as individual values with means and standard deviations. Statistical analysis was performed using the Wilcoxon test. *p < 0.05.

Figure 3 presents the relationships between mature TLS and intratumoral immune cell infiltration, including T cells (CD3, CD8, FOXP3), macrophages (CD86, CD163), and CD20^+^ B cells. Primary tumors with mature TLS showed higher levels of intratumoral CD3^+^ and CD8^+^ T lymphocyte infiltration than those without mature TLS (Figure 3A).

Among 78 patients with advanced CRC, metastatic lesions in 11 patients (liver metastasis in seven cases and lung metastasis in four cases) were resected before the initiation of systemic chemotherapy. The CD3^+^ and CD8^+^ T lymphocyte infiltration within these lesions was evaluated to demonstrate the relationship between mature TLS status in the primary tumor and intratumoral T lymphocyte infiltration in metastatic lesions. Mature TLS‐positive cases in the primary tumor exhibited higher levels of intratumoral CD3^+^ and CD8^+^ T lymphocyte infiltration not only in the primary site but also in the corresponding metastatic lesions (Figure 3B,C). These findings suggest that TLS maturity in untreated primary tumors prior to systemic chemotherapy is closely associated with tumor immunity at therapeutic target lesions in metastatic organs.

## 4 Discussion

This study demonstrated that mature TLS in primary tumors of patients with metastatic or recurrent CRC prior to systemic chemotherapy is significantly associated with a favorable chemotherapy response and longer survival. Multivariate analysis confirmed TLS maturity as an independent predictor of both therapeutic response and survival outcomes in this cohort. TLS maturity was also closely associated with the presence of MAdCAM‐1–positive vessels, which facilitate lymphocyte recruitment, as well as with distinct patterns of tumor‐infiltrating immune cell composition, including higher levels of CD3^+^ and CD8^+^ T‐cell infiltration not only in primary tumors but also in matched metastatic lesions. To our knowledge, this is the first report to demonstrate the potential of TLS maturity in pretreatment primary tumors as a biomarker for treatment sensitivity, prognosis, and systemic tumor immunity in advanced CRC.

A close relationship between systemic chemotherapy and tumor immunity has been previously reported. Two major mechanisms are involved in tumor control by cytotoxic agents: direct killing of cancer cells by chemotherapy and secondary tumor cell elimination mediated by the host immune system. In the latter mechanism, tumor antigens delivered from chemotherapy‐damaged cancer cells are taken up by dendritic cells, which then migrate to regional lymph nodes and induce tumor‐specific T cells. These T cells subsequently migrate to the tumor site and exert cytotoxic activity against cancer cells [15]. Notably, previous studies have shown that the efficacy of chemotherapy is markedly reduced in immunodeficient mice [16], and that tumors with abundant CD3^+^ and CD8^+^ tumor‐infiltrating lymphocytes, referred to as hot tumors, are more likely to achieve a pathological CR following neoadjuvant chemotherapy [17].

The recruitment and activation of immune cells in the tumor microenvironment are essential in determining chemotherapy sensitivity. TLS has also been reported to regulate the accumulation and activation of immune cells, including T cells, within the tumor microenvironment [18, 19]. Consistent with these reports, our study demonstrated that mature TLS positivity in advanced CRC was associated with a favorable response to systemic chemotherapy and higher levels of CD3^+^ and CD8^+^ lymphocyte infiltration in both primary and metastatic tumors. These findings suggest that the enhanced chemotherapy sensitivity observed in the mature TLS‐positive group results from secondary activation of host anti‐tumor immunity mediated by TLS.

Previous studies have reported that the presence of mature TLS is associated with improved response rates and OS following immune checkpoint inhibitor therapy in cancers, including lung, bladder, breast, and renal [20]. With respect to chemotherapy, prior investigations have predominantly assessed the effect of neoadjuvant treatment, demonstrating an association between TLS within the primary tumor and the local therapeutic response. Specifically, in esophageal and lung cancers, mature TLS have been reported to correlate with response to neoadjuvant chemotherapy [8, 21]. Consistent with these findings in other malignancies, the results of the present study suggest that TLS are also linked to chemotherapy efficacy in CRC from the perspective of tumor immunity. A novel and noteworthy observation of our study is that TLS maturity in the primary tumor, assessed prior to systemic therapy, may predict systemic chemotherapy response even in recurrent or distant metastatic disease. Notably, this is the first report using clinical cancer specimens to demonstrate a significant association between TLS maturity in primary tumors and intratumoral T lymphocyte infiltration levels at metastatic sites. Therefore, we explored the potential mechanisms linking TLS maturity in primary tumors with tumor immune status in both primary and recurrent/metastatic lesions, particularly in terms of T‐cell infiltration. In cases where synchronous distant metastases were the target of chemotherapy, the presence of mature TLS in the primary tumor may have contributed to the activation of local anti‐tumor immunity. Specifically, mature TLS can efficiently induce cancer‐specific T cells by causing antigen presentation and T‐cell activation in the vicinity of the tumor [22]. These activated T cells may circulate systemically and reach distant metastatic lesions, contributing to a broader anti‐tumor immune response. In recurrent cases, the presence of mature TLS in the primary tumor may have facilitated the induction and maintenance of memory T cells prior to recurrence, in contrast to tumors with immature TLS. Previous studies have shown that the presence of TLS is associated with the accumulation of memory T cells, which are critical for regulating immune responses upon tumor recurrence [23, 24]. Moreover, memory T cells have been reported to rapidly differentiate into effector T cells and be recruited to recurrent lesions during progression from micrometastasis to overt macrometastasis [25, 26], suggesting the importance of memory T cells for significant anti‐tumor immunity in TLS‐mature positive cases. Therefore, our findings suggest that evaluating TLS maturity in archived primary tumors at surgery may provide a useful indicator for predicting treatment sensitivity and tumor immune status in patients with advanced CRC, even before initiating systemic therapy for recurrent or metastatic disease.

In this study, we found that mature TLSs were surrounded by a greater number of MAdCAM‐1‐positive vessels than immature TLSs. The formation of high endothelial venules (HEVs), which connect TLS to the bloodstream and enable sustained lymphocyte recruitment, is critical for TLS maturation [7]. Indeed, previous studies have suggested that HEVs contribute to the formation and maturation of TLS [27], and that higher densities of HEVs and TLS are associated with favorable clinical outcomes [14]. MAdCAM‐1 is expressed on HEV and contributes to lymphocyte recruitment through α4β7 interaction [28]. Our findings suggest that MAdCAM‐1 expression may also be closely involved in the formation and maturation of TLS in advanced CRC. Additionally, several studies have shown that lymphoid chemokines, including CXCL13, play an important role in regulating TLS development [7, 29, 30]. In a murine model of pancreatic cancer, intratumoral administration of CXCL13 and CCL21 was shown to induce TLS formation. Furthermore, combining systemic chemotherapy (gemcitabine) with intratumoral delivery of lymphoid chemokines enhanced TLS formation and led to significant tumor regression, which was not achieved with chemotherapy alone [29]. Therefore, it may be promising to explore therapeutic strategies that enhance the expression of CXCL13 or promote the development of MAdCAM‐1–positive vessels to induce mature TLS formation. Such approaches may help convert the tumor immune microenvironment of advanced CRC into a hot tumor phenotype, potentially improving the efficacy of chemotherapy and immune checkpoint blockade therapy.

This study has some limitations. First, it was a single‐center retrospective observational study with a limited number of cases. Further validation in larger cohorts through multi‐center collaborative studies will be necessary. Second, TLS maturity was assessed based on pathological analysis of a 1‐mm region from the invasive front of the primary tumor. This method is inapplicable to biopsy specimens and may, therefore, be unsuitable for patients with unresectable advanced CRC. Third, the significant association observed in this study between TLS maturity and efficacy of cytotoxic chemotherapy remains mechanistically unclear. Functional analyses of TLS maturity using in vitro or in vivo models were not performed in this study, and further investigation is needed to clarify the underlying mechanisms.

## 5 Conclusions

Our findings demonstrate that TLS maturity in pretreatment primary tumors is associated with chemotherapy response, survival outcomes, and tumor immune profiles in patients with metastatic or recurrent CRC. The consistent association between TLS maturity and immune cell infiltration in both primary and metastatic sites emphasizes the relevance of TLS maturity to systemic tumor immunity. These results suggest that TLS maturity in primary tumors may be a novel biomarker for predicting systemic chemotherapy response and immune status across primary and metastatic sites in advanced CRC.

## Author Contributions

**Nobuhiro Hosoi:** writing – original draft, data curation, project administration, writing – review and editing, investigation. **Chika Komine:** funding acquisition, investigation. **Gendensuren Dorjkhorloo:** conceptualization, data curation. **Takuya Shiraishi:** project administration, supervision. **Takuhisa Okada:** data curation. **Akihiko Sano:** data curation. **Makoto Sakai:** data curation. **Takehiko Yokobori:** supervision, project administration. **Ken Shirabe:** supervision, project administration. **Hiroshi Saeki:** supervision, project administration.

## Funding

This study was supported by Grants‐in‐Aid for Scientific Research from the JSPS (24 K23373, 24 K23504, and 25 K19724).

## Ethics Statement

The protocol for this research project has been approved by a suitably constituted Ethics Committee of the institution, and it conforms to the provisions of the Declaration of Helsinki. Committee of the Graduate School of Medicine, Gunma University, Approval No. HS2024‐072. Informed consent was obtained through an opt‐out on the website.

## Conflicts of Interest

Authors Ken Shirabe and Hiroshi Saeki are editorial board members of Annals of Gastroenterological Surgery. Other authors declare no conflicts of interest for this article.

## Supporting information


**Figure S1:** Kaplan–Meier survival curves of patients with CRC stratified by TLS maturity for each metastatic lesion.
Kaplan–Meier analyses of PFS in patients with liver metastasis (*p* = 0.0155, upper left panel), lung metastasis (*p* = 0.0004, upper right panel), and peritoneal dissemination (*p* = 0.0245, lower left panel). *p < 0.05. Statistical significance was determined using the log‐rank test.

**Table S1:** Patient Characteristics.
