# Supplementary material for: Mature Tertiary Lymphoid Structures Indicate Good Chemotherapy Response and Prognosis in Advanced Colorectal Cancer
Source: Ann Gastroenterol Surg. 2025 Dec 2;10(3):710–21. doi: 10.1002/ags3.70142 (PMC13178286; doi:10.1002/ags3.70142)
Supplement: Supplementary file 1 — Figure S1: Kaplan–Meier survival curves of patients with CRC stratified by TLS maturity for each metastatic lesion. Kaplan–Meier analyses of PFS in patients with liver metastasis (p = 0.0155, upper left panel), lung metastasis (p = 0.0004, upper right panel), and peritoneal dissemination (p = 0.0245, lower left panel). *p < 0.05. Statistical significance was determined using the log‐rank test. [file AGS3-10-710-s001.pdf]

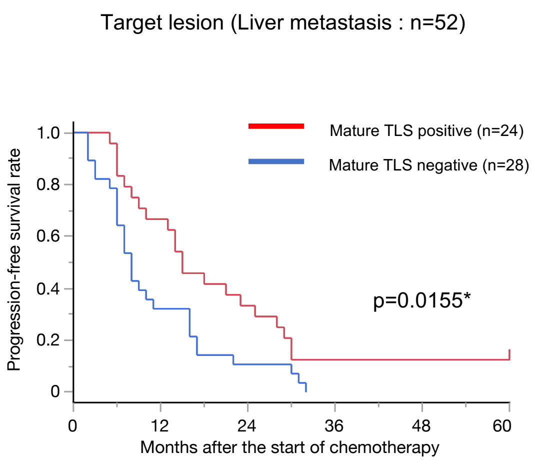
Target lesion (Liver metastasis : n=52)
Mature TLS positive (n=24)
Mature TLS negative (n=28)
p=0.0155*
Progression-free survival rate
1.0
0.8
0.6
0.4
0.2
0
0
12
24
36
48
60
Months after the start of chemotherapy

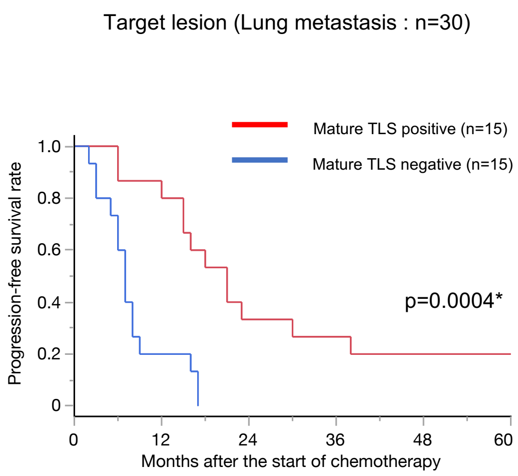
Target lesion (Lung metastasis : n=30)
Mature TLS positive (n=15)
Mature TLS negative (n=15)
p=0.0004*
Progression-free survival rate
1.0
0.8
0.6
0.4
0.2
0
0
12
24
36
48
60
Months after the start of chemotherapy

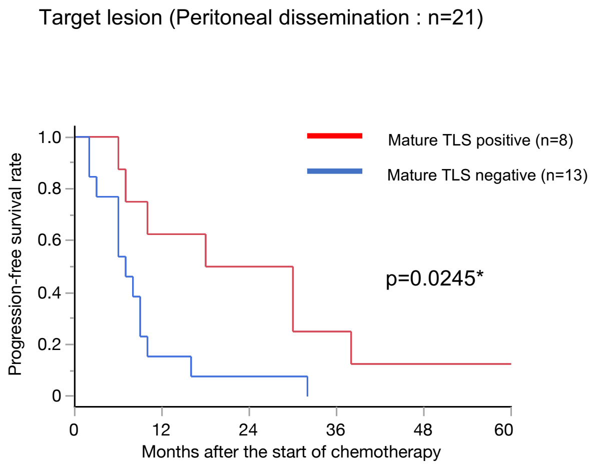
Target lesion (Peritoneal dissemination : n=21)
Mature TLS positive (n=8)
Mature TLS negative (n=13)
p=0.0245*
Progression-free survival rate
1.0
0.8
0.6
0.4
0.2
0
0
12
24
36
48
60
Months after the start of chemotherapy
