# Supplementary material for: Mature Tertiary Lymphoid Structures Indicate Good Chemotherapy Response and Prognosis in Advanced Colorectal Cancer
Source: Ann Gastroenterol Surg. 2025 Dec 2;10(3):710–21. doi: 10.1002/ags3.70142 (PMC13178286; doi:10.1002/ags3.70142)
Supplement: Supplementary file 2 — Table S1: Patient Characteristics. [file AGS3-10-710-s002.docx]

**Supporting information**

**Supplementary Table S1**

Patient Characteristics

| Characteristics | Mature TLS | | p-value |
| --- | --- | --- | --- |
|  | Negative  (n=43) | Positive  (n=35) |  |
| Stage |  |  |  |
| Ⅰ, Ⅱ | 5 (11.6) | 4 (11.4) | 0.9686 |
| Ⅲ | 10 (24.4) | 9 (25.7) |  |
| Ⅳ | 28 (64) | 22 (62.9) |  |
| Metastasis lesion |  |  |  |
| Liver |  |  |  |
| Absence | 15 (34.9) | 11 (31.4) | 0.7475 |
| Presence | 28 (65.1) | 24 (68.6) |  |
| Lung |  |  |  |
| Absence | 28 (65.1) | 20 (57.1) | 0.4716 |
| Presence | 15 (34.9) | 15 (42.9) |  |
| Peritoneal dissemination |  |  |  |
| Absence | 30 (69.8) | 27 (77.1) | 0.4652 |
| Presence | 13 (30.2) | 8 (22.9) |  |
| Tumor marker |  |  |  |
| CEA (mean (SD)) | 206.1 (544.2) | 54.9 (108.5) | 0.6694 |
| CA19-9 (mean (SD)) | 248.7 (816.3) | 66.7 (197.4) | 0.1811 |
| Complications |  |  |  |
| Hypertension |  |  |  |
| Absence | 27 (62.8) | 21 (60) | 0.8011 |
| Presence | 16 (37.2) | 14 (40) |  |
| Diabetes mellitus |  |  |  |
| Absence | 29 (67.4) | 28 (80) | 0.2136 |
| Presence | 14 (32.6) | 7 (20) |  |
| Chronic obstructive pulmonary disease |  |  |  |
| Absence | 39 (83.7) | 33 (94.3) | 0.5542 |
| Presence | 4 (16.3) | 2 (5.7) |  |
| RAS mutation status (n=67) |  |  |  |
| Wild | 17 (47.2) | 16 (51.6) | 0.7200 |
| Mutant | 19 (52.8) | 15 (48.4) |  |
| BRAF mutation status (n=16) |  |  |  |
| Wild | 5 (83.3) | 9 (90) | 1.0000 |
| Mutant | 1 (16.7) | 1 (10) |  |
| Microsatellite status (n=15) |  |  |  |
| MSS | 2 (66.7) | 12 (100) | 0.2000 |
| MSI | 1 (33.3) | 0 (0) |  |

TLS, tertiary lymphoid structure; SD*,* standard deviation; CEA, carcinoembryonic antigen; CA19-9, carbohydrate antigen 19-9; MSS, microsatellite stable; MSI, microsatellite instability
